# Physician Perspectives on Pharmaceutical Promotion

**DOI:** 10.1001/jamahealthforum.2025.3521

**Published:** 2025-09-05

**Authors:** Helen Mooney, Kirsten Austad, Eric G. Campbell, Jerry Avorn, Zhigang Lu, Aaron S. Kesselheim

**Affiliations:** 1 Program on Regulation, Therapeutics, and Law (PORTAL), Division of Pharmacoepidemiology and Pharmacoeconomics, Department of Medicine, Brigham and Women’s Hospital, Boston, Massachusetts; 2 Department of Family Medicine, Boston Medical Center & Boston University Chobanian and Avedisian School of Medicine, Boston, Massachusetts; 3 Evans Center for Implementation and Improvement Sciences, Department of Medicine, Boston University Chobanian and Avedisian School of Medicine, Boston, Massachusetts; 4 University of Colorado Anschutz School of Medicine, Department of Medicine, Division of General Internal Medicine and Center for Bioethics and Humanities, Aurora; 5 Harvard Medical School, Boston, Massachusetts

## Abstract

This survey study reports the responses of medical students and residents about their interactions with and attitudes toward the pharmaceutical industry and changes over 13 years, from 2011 to 2024.

## Introduction

Pharmaceutical manufacturers spend over $35 billion per year marketing drugs, often directly to health care professionals.^1^^(p295)^ In 2011, we surveyed medical students and residents about their interactions with and attitudes toward the pharmaceutical industry.^2,3,4,5^ We then resurveyed the same trainees 13 years later to investigate changes in attitudes toward the pharmaceutical industry.

## Methods

To assemble respondents in 2011, we randomly selected US medical students and residents. Of the 3495 students and 1815 residents surveyed, 1610 medical students (49%) and 739 residents (43%) responded.^2,3,4,5^ In 2024, we collected emails for 1130 of the respondents from the 2011 study and supplemented them with publicly available email addresses. In both surveys, we asked participants to describe their interactions with and attitudes towards the pharmaceutical industry. This study was approved by the Mass General Brigham institutional review board and aligns with the AAPOR reporting guideline. The 2024 survey questions and design, administration, and analysis details are provided in the eAppendix and eMethods in Supplement 1.

## Results

From the initial respondents, 229 recontact emails were invalid, yielding 901 eligible potential respondents. Of these, 294 responded (33% response rate) and 291 were matched to their 2011 responses. The mean (SD) age of respondents was 40.8 (3.8) years; 49.5% of participants identified as women. Most respondents were medical students in 2011 (72.9%) and did not attend top 20 National Institutes of Health–funded medical schools (74.1%). Respondents were most likely to report working in hospitals or clinics (74.2%), followed by academic research institutions (29.9%), medical schools (27.2%), and private practice (24.4%). Most reported caring for patients for more than half of their time (80.4%) and were specialists (70.9%) (Table).

**Table.** Characteristics of 291 Participants Who Were Matched to Their 2011 Responses

Characteristic	Participants, No. (%)
Age, mean (SD), y	40.8 (3.8)
Gender^a^	
Man	141 (48.5)
Woman	144 (49.5)
Other^b^	6 (2.1)
Race and ethnicity^c^	
Asian	60 (20.6)
Black or African American	16 (5.5)
Hispanic or Latino	14 (4.8)
White	209 (71.8)
Other^d^	11 (3.8)
Institution^c^	
Hospital/clinic	216 (74.2)
Academic research institution	87 (29.9)
Medical school	79 (27.2)
Private practice	71 (24.4)
Government agency	31 (10.7)
Other	12 (4.1)
Pharmaceutical company/industry	3 (1.0)
Work in the direct care of patients	
0%	8 (2.8)
1%-9%	4 (1.4)
10%-24%	14 (4.8)
25%-49%	31 (10.7)
50%-100%	234 (80.4)
Type of practice	
Primary care	84 (29.1)
Specialty care	205 (70.9)
2011 Status^e^	
Resident	79 (27.2)
Medical student	212 (72.9)
School type	
Top 20 NIH-funded medical school	75 (26.0)
Other medical school	214 (74.1)
Receipt of gifts	
2011 Gift/2024 no gift	55 (19.0)
2011 No gift/2024 gift	63 (21.8)
2011 No gift/2024 no gift	98 (33.9)
2011 Gift/2024 gift	73 (25.3)

Abbreviation: NIH, National Institutes of Health.

^a^ In the 2011 survey (before the updates about gender identity), 1112 (50.9%) responded that they identified as male and 1069 (48.9%) identified as female.^2^

^b^ Includes participants who answered nonbinary or prefer not to say.

^c^ Nonexclusive responses. Race and ethnicity were ascertained from the 2024 survey question asking respondents to choose 1 or more race and ethnicity categories from a list. The question was asked to determine the representativeness of the respondents.

^d^ Includes participants who answered Native Hawaiian or Pacific Islander, or Native American, or other.

^e^ In the 2011 survey, 1610 (68.5%) respondents were medical students and 739 (31.5%) were residents.^2^

On follow-up, respondents were more likely to strongly agree that trust in medicine was threatened by industry marketing interactions (5.6% in 2011 vs 14.5% in 2024; *P* = .004) but also that physicians can receive useful information from marketing (66.3% in 2011 vs 76.9% in 2024; *P* = .005). There were no shifts in perceptions that interactions with pharmaceutical representatives lead to more free drug samples for patients in need, to increased use of more expensive drugs without additional benefit, or to creating unconscious bias in favor of sponsors’ products. The perception that limits on pharmaceutical consulting can hamper drug development became slightly more common (23.1% in 2011 vs 28.5% in 2024; *P* = .01). We found increased agreement that medical schools should not permit interactions between industry and students in training facilities (59.6% in 2011 vs 72.2% in 2024; *P* = .004) and greater agreement that schools should require faculty to disclose conflicts of interest before lectures (88.9% in 2011 vs 99.7% in 2024; *P* < .001) (Figure).

**Figure.**
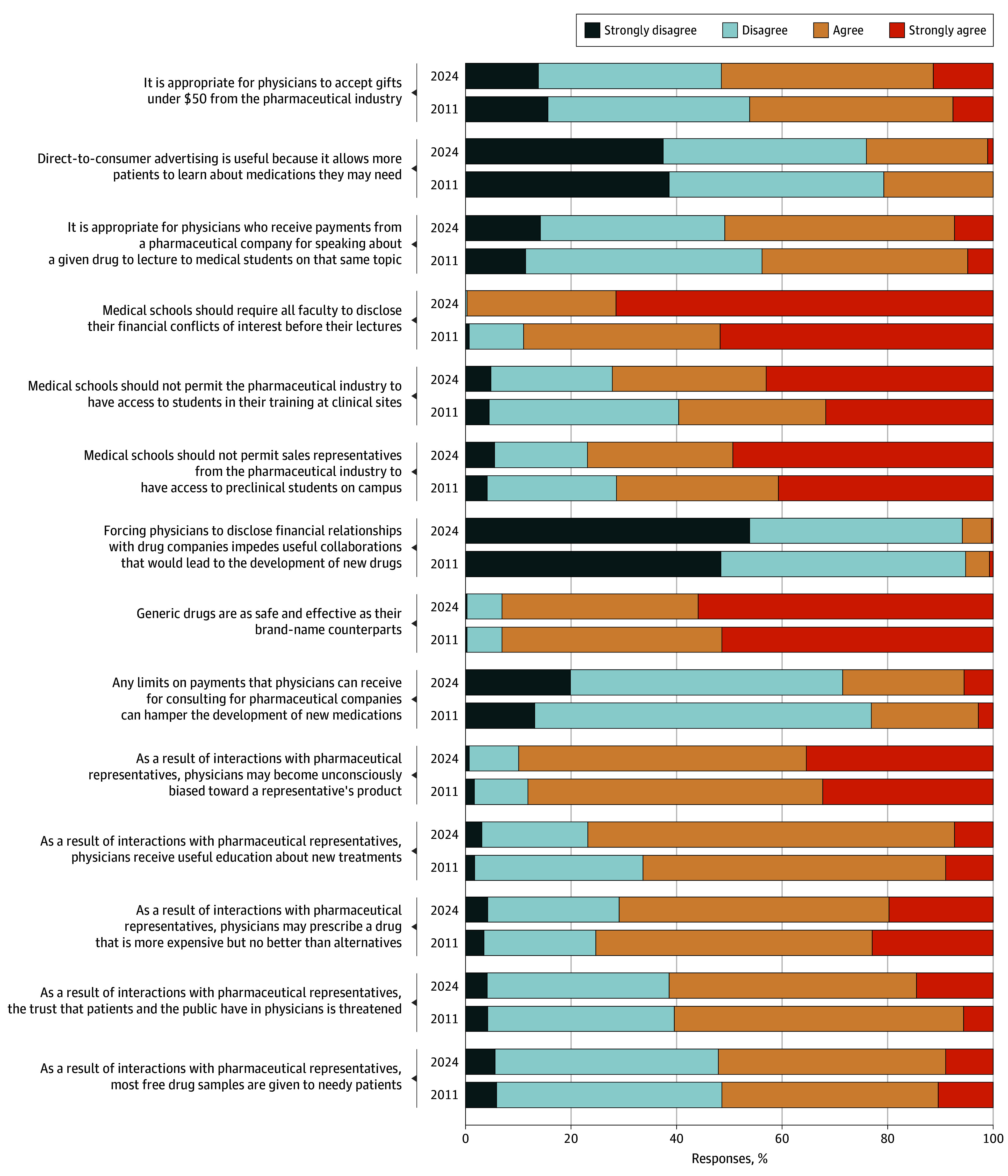
Changes Over Time in Perceptions of Pharmaceutical Promotion from Trainees (2011) to Physician Professionals (2024)

## Discussion

This 13-year longitudinal survey study found that physician perceptions of industry evolved, with increasing confidence in industry information about drugs but growing concern about the role of industry-physician marketing interactions in trust in medicine. Shifts in attitude may be attributable to respondents’ leaving training and assuming professional roles or may represent secular changes over time. The 13-year timeline and survey methodology limited our response rate and led to an oversampling of academic researchers. Our survey also relies on self-reporting, and participants might have reported less favorable attitudes toward industry due to social desirability bias.

Despite these challenges, this interval survey provides further evidence of physician concern about and reliance on pharmaceutical marketing. Researchers in diverse settings over multiple decades have repeatedly found direct associations between pharmaceutical detailing, prescribing choices,^5^ and ultimately patient outcomes.^6^ Professional societies, payers, and regulators should collaborate to provide alternatives to drug marketing to early-career and midcareer physicians, who may welcome such opportunities even more so than when they were trainees.

## Notes

Supplement 1. **eAppendix.** Relevant Survey Questions **eMethods.** Survey Sample, Survey Design, Survey Administration, Data Analysis
eReferences

Supplement 2.
Data Sharing Statement
