# Supplementary material for: Physician Perspectives on Pharmaceutical Promotion
Source: JAMA Health Forum. 2025 Sep 5;6(9):e253521. doi: 10.1001/jamahealthforum.2025.3521 (PMC12413643; doi:10.1001/jamahealthforum.2025.3521)
Supplement: Supplement 1. — eAppendix. Relevant Survey Questions eMethods. Survey Sample, Survey Design, Survey Administration, Data Analysis eReferences [file jamahealthforum-e253521-s001.pdf]

Supplemental Online Content

Mooney H, Austad K, Campbell EG, Avorn J, Lu Z, Kesselheim AS. Physician perspectives on pharmaceutical promotion. *JAMA Health Forum.* 2025;6(9):e253521. doi:10.1001/jamahealthforum.2025.3521

eAppendix. Relevant Survey Questions

eMethods. Survey Sample, Survey Design, Survey Administration, Data Analysis

eReferences

This supplemental material has been provided by the authors to give readers additional information about their work.

© 2025 Mooney H et al. *JAMA Health Forum*.

eAppendix. Relevant Survey Questions

How much do you agree or disagree that as a result of physicians meeting with pharmaceutical representatives…				
	Strongly agree	Agree	Disagree	Strongly disagree
Most free drug samples are given to needy patients				
The trust that patients and the public have in physicians is threatened				
Physicians may prescribe a drug that is more expensive but no better than alternatives				
Physicians receive useful education about new treatments				
Physicians may become unconsciously biased toward a representative's product				

**How much do you agree or disagree with the following statements?**	**Strongly agree**	**Agree**	**Disagree**	**Strongly disagree**
Any limits on payments that doctors can receive for consulting for pharmaceutical companies can hamper the development of new medications				
Generic drugs are as safe and effective as their brand-name counterparts				
Forcing physicians to disclose financial relationships with drug companies impedes useful collaborations that would lead to the development of new drugs				
Medical schools should not permit sales representatives from the pharmaceutical industry to have access to preclinical students on campus				
Medical schools should not permit the pharmaceutical industry to have access to students in their training at clinical sites				
Medical schools should require all faculty to disclose their financial conflicts of interest before their lectures				
It is appropriate for physicians who receive payments from a pharmaceutical company for speaking about a given drug to lecture to medical students on that same topic				
Direct-to-consumer advertising is useful because it allows more patients to learn about medications they may need				
It is appropriate for physicians to accept gifts under $50 from the pharmaceutical industry				

© 2025 Mooney H et al. *JAMA Health Forum*.

Demographic questions and medical practice characteristics:

What is your gender?	Man<br>Woman<br>Non-Binary<br>Prefer not to say

Choose one or more races/ethnicities you identify as:	American Indian/Alaskan Native<br>Asian<br>Black/African American<br>Hispanic/Latino<br>Native Hawaiian or Pacific Islander<br>White<br>Other

What percentage of your work is in the direct care of patients?	0%<br>1-9%<br>10-24%<br>25-49%<br>50%-100%

What type of medicine do you practice?	Primary Care<br>Specialty Care

What institution(s) do you work for? Please check all that apply:	Hospital/clinic<br>Private practice<br>Medical school<br>Academic research institution<br>Government agency<br>Pharmaceutical company/industry<br>Other

Have you received any of the following in the last six months from drug, device or other medically related companies?		
	Yes	No
Food or beverage in the workplace		
Free drug samples		
A meal outside of the office, campus, or hospital		
Pens, notepads, T-shirts		
Any other gift or financial support		

eMethods. Survey Sample, Survey Design, Survey Administration, Data Analysis

Survey Sample

© 2025 Mooney H et al. *JAMA Health Forum*.

We sought to resurvey participants who responded to our 2011 national survey of medical students and residents.[1-4] To establish the initial survey cohort in 2011, our team used the American Medical Association's Physician Masterfile. That survey excluded trainees in osteopathic medicine, international students, and members of the armed services, as well as those with incomplete contact information. For the initial study, we stratified the remaining 16,299 first-year students, 14,804 fourth-year students, and 14,266 third-year residents by medical school. For each medical school, we selected a random sample of 14 first-year students, 15 fourth-year students, and 15 residents, yielding 3,495 medical students and 1,815 residents, of whom 1,610 medical students (49% response rate) and 739 residents (43% response rate) participated.[1-4] For the 2024 follow-up study, we found 1,130 email addresses using responses from the earlier study's paper records and Excel data files.

Survey Design

Given our intent to study changes in attitudes and behaviors over time, our survey instrument started with the same 2011 questionnaire that asked participants to describe their perceptions of medical practice and industry. We made minor changes to reflect respondents' current career status. Respondents were asked to report whether they had received gifts, drug samples, meals, or other financial support from industry within the last six months. We then asked several questions concerning participants' attitudes toward the drug industry and medical practice. Respondents were asked to indicate the appropriateness of gifts, consulting, and educational materials, the likelihood that gifts change prescribing practices, and the impact of such interactions on patient care. Respondents were also asked to indicate their feelings toward limits on direct-to-consumer advertising, generic drugs, and the impact of industry interactions on medical education. Given that all participants responded to the 2011 survey as a prerequisite for 2024 participation, we had records of previously-reported demographic information. We asked updated demographic questions regarding gender, race/ethnicity, type of patient care and practice, and institutional base. This survey was approved by the Mass General Brigham Institutional Review Board.

Survey Administration

The survey was administered electronically using REDCap software. After three email solicitations, we searched the Internet for publicly-available contact information for non-respondents. We sought out current contact information for participants whose emails delivered but had not responded to the survey, yielding 72 new email addresses. We sent a total of six waves of emails. Participants received $50 Amazon gift cards for completing the survey.

Data Analysis

We matched 2024 responses to 2011 responses using individual study ID numbers assigned in 2011. We categorized respondents by age, gender, race/ethnicity, patient institution type, percentage of time spent in direct patient care, type of practice, training status in 2011, medical school ranking, and reported receipt of gifts. To categorize respondents by educational institution, we labeled each participant's medical school with its 2011 NIH funding ranking.[5] We stratified responses based on whether participants attended top 20 or non-top 20 medical schools by this measure. We also categorized responses by whether participants reported receiving gifts from the pharmaceutical industry in 2011 and/or 2024.

We performed chi-square tests to determine changes in attitudes towards the pharmaceutical industry between 2011 and 2024. These questions asked about respondents'

© 2025 Mooney H et al. *JAMA Health Forum*.

opinions about interactions between clinicians and industry and the perceived impact of interactions with pharmaceutical representatives. All statistical analyses were performed using SAS 9.4 software (SAS Institute, NC). The level of statistical significance was set at $p < 0.05$.

Informed Consent

Consent to participate was inferred from survey participation. The survey's introduction included the following statement: "your participation in this survey is voluntary, and you may withdraw at any time."

eReferences

[1] Austad KE, Avorn J, Franklin JM, Kowal MK, Campbell EG, Kesselheim AS. Changing interactions between physician trainees and the pharmaceutical industry: a national survey. *J Gen Intern Med*. 2013;28(8):1064-1071.

[2] Yeh JS, Austad KE, Franklin JM, et al. Association of medical students' reports of interactions with the pharmaceutical and medical device industries and medical school policies and characteristics: a cross-sectional study. *PLoS Medicine* 2014;11(10):e1001743.

[3] Yeh JS, Austad KE, Franklin JM, et al. Medical Schools' Industry Interaction Policies Not Associated With Trainees' Self-Reported Behavior as Residents: Results of a National Survey. *J Grad Med Educ* 2015;7(4):595-602.

[4] Austad KE, Avorn J, Franklin JM, Campbell EG, Kesselheim AS. Association of marketing interactions with medical trainees' knowledge about evidence-based prescribing: results from a national survey. *JAMA Intern Med*. 2014;174(8):1283-1290.

[5] BRIMR Rankings of NIH Funding in 2011 | BRIMR. March 21, 2023. Accessed October 30, 2024. https://brimr.org/brimr-rankings-of-nih-funding-in-2011/

© 2025 Mooney H et al. *JAMA Health Forum*.
