# Supplementary material for: Physician Perspectives on Pharmaceutical Promotion
Source: JAMA Health Forum. 2025 Sep 5;6(9):e253521. doi: 10.1001/jamahealthforum.2025.3521 (PMC12413643; doi:10.1001/jamahealthforum.2025.3521)
Supplement: Supplement 2. — Data Sharing Statement [file jamahealthforum-e253521-s002.pdf]

Data Sharing Statement

Mooney. Physician Perspectives on Pharmaceutical Promotion. *JAMA Health Forum*.
Published September 05, 2025. doi:10.1001/jamahealthforum.2025.3521

Data
Data available: No
